# Glymphatic system dysfunction in children with autism spectrum disorder as evidenced by the diffusion tensor imaging along perivascular spaces index

**DOI:** 10.3389/fpsyt.2025.1701816

**Published:** 2025-10-29

**Authors:** Shengnan Zhao, Manxue Zhang, Tingting Luo, Lei Li, Yuchu Jiang, Mingjing Situ, Yi Huang

**Affiliations:** ^1^ Mental Health Center, West China Hospital, Sichuan University, Chengdu, Sichuan, China; ^2^ Laboratory of Child and Adolescent Psychiatry, Mental Health Center and Psychiatric Laboratory, West China Hospital, Sichuan University, Chengdu, Sichuan, China; ^3^ Child Mental Health Center, West China Hospital, Sichuan University, Chengdu, Sichuan, China

**Keywords:** autism spectrum disorder, glymphatic system, diffusion tensor imaging along perivascular spaces (DTI-ALPS), visual-motor integration (VMI) function, communication deficits

## Abstract

**Introduction:**

This study aims to evaluate glymphatic system function in autism spectrum disorder (ASD) by employing diffusion tensor imaging along perivascular spaces (DTI-ALPS), and investigate its relationship with visual-motor integration (VMI) function.

**Materials and methods:**

A total of 78 individuals with ASD and 48 typically developing (TD) children were enrolled. All participants underwent diffusion tensor imaging on a 3-T MRI scanner. The DTI-ALPS index was calculated, and data on IQ and VMI function were obtained. Independent-samples t-test was used to compare the DTI-ALPS index between groups. Correlation analysis was conducted to examine the relationships between the DTI-ALPS index and clinical variables, including core symptoms, within the ASD group. Mediation analysis explored the relationship among the DTI-ALPS index, core symptoms, and VMI function.

**Results:**

Compared to the TD group, ASD patients showed significantly reduced DTI-ALPS indices in the left hemisphere (DTI-ALPS-L), right hemisphere (DTI-ALPS-R), and for the whole-brain mean (Mean DTI-ALPS). In the ASD group, these indices were negatively correlated with the Autism Diagnostic Interview-Revised (ADI_R) communication score but positively correlated to the VMI score. Mediation analysis revealed that the VMI score significantly mediated the relationship between DTI-ALPS-R and the ADI_R communication score (indirect effect β = -0.082, p< 0.001).

**Conclusions:**

Our preliminary findings indicate impaired glymphatic system function in ASD, which may contribute to its pathogenesis. Furthermore, VMI function mediates the relationship between altered glymphatic system function and communication deficits in ASD.

## Introduction

Autism spectrum disorder (ASD) is a common neurodevelopmental disorder characterized by social communication/interaction and restrictive, repetitive patterns of behavior (RRB) (1). In recent years, the prevalence of ASD has increased globally, with current estimates at approximately 1% (2). This increasing prevalence places a substantial psychological and economic burden on families and society. However, the pathogenesis of ASD remains unclear. Therefore, elucidating the etiology and pathogenesis of ASD is crucial to provide a theoretical basis for its biological diagnosis and precise treatment.

The glymphatic system is a crucial pathway for the clearance of metabolic waste from the brain. Its structural foundation is a network of perivascular spaces (PVS) formed by the perivascular end-feet of astrocytes surrounding blood vessels. Cerebrospinal fluid (CSF) and interstitial fluid (ISF) circulate along these channels, facilitating solute exchange and thereby maintaining cerebral microenvironmental homeostasis (3, 4). Diffusion tensor imaging analysis along the perivascular space (DTI-ALPS) is a recently proposed, non-invasive technique for evaluating glymphatic system function. This technique quantifies the anisotropy of water diffusion along perivascular pathways without the need for contrast enhancement; the resulting ALPS index serves as a direct biomarker of glymphatic activity and has been widely applied to assess glymphatic dysfunction in various neurological disorders (5, 6). Accumulating evidence indicates that impaired glymphatic function constitutes an important pathophysiological mechanism underlying Alzheimer’s disease (7), Parkinson’s disease (8), and other neurodegenerative conditions, and is closely associated with deficits in cognition and memory (9). More recently, the role of the glymphatic system in psychiatric disorders such as major depressive disorder (10) and schizophrenia (11) has garnered increasing attention. For instance, Tu et al. reported significantly reduced ALPS indices in patients with schizophrenia, which correlated with cognitive impairment, suggesting that compromised glymphatic function may represent a potential pathophysiological substrate of the disorder (12). Using the DTI-ALPS method, Li et al. observed bilateral decreases in ALPS indices in individuals with ASD (13). However, their study was limited by a small sample size and solely compared ALPS indices between ASD patients and healthy controls without exploring associations with clinical features, thereby restricting the generalizability of the findings. Therefore, further investigation is warranted.

Children with ASD exhibit atypical visual-motor integration (VMI), manifesting as heightened variability during visually guided precision movements, a phenomenon attributed to insufficient visual-feedback control of motor output (14). VMI denotes the capacity to link visual stimuli with appropriate and accurate motor responses, reflecting the coordinated interaction between visual perception and motor execution (15). Furthermore, children with ASD display marked impairments in eye-hand coordination, including deficient goal-directed control of aiming movements (16); this coordination represents a core element of VMI. As a fundamental aspect of neurodevelopment, VMI is indispensable for acquiring the skills required in daily learning, occupational tasks, and routine activities (17). Sensory integration training is an evidence-based intervention that has been shown to enhance cognitive function in children with ASD (18, 19). Nevertheless, VMI in ASD remains understudied. Although previous work has demonstrated early VMI deficits in Parkinson’s disease (20), and motor symptoms in this disorder correlate with reduced ALPS indices (21, 22), the underlying pathophysiological mechanisms likely differ from those in neurodevelopmental disorders such as ASD. To the best of our knowledge, the relationship between glymphatic system function and VMI in ASD has not been established.

We hypothesize that glymphatic system function is impaired in individuals with ASD and that this impairment is associated with deficits in VMI. Using the DTI-ALPS method, this study aims to evaluate glymphatic system function in children with ASD and to elucidate its relationship with VMI performance, thereby providing novel insights into the role of cerebral waste-clearance mechanisms in ASD pathophysiology.

## Materials and methods

### Population

This study was designed as a case-control investigation. Participants were recruited, screened, and assessed at West China Hospital of Sichuan University between October 2022 and October 2023. The research protocol was approved by the Biomedical Ethics Committee of West China Hospital (IRB No. 2022-1402). Written informed consent was obtained from all parents after they received a detailed description of the study, and all data were used strictly for research purposes.

Eligibility criteria for the ASD group included children aged 3 to 18 years who were diagnosed by a licensed child psychiatrist according to the DSM-5 criteria. Exclusion criteria were as follows: (1) presence of neurological disorders (e.g., epilepsy or encephalitis); (2) history of significant craniocerebral trauma; (3) known monogenetic syndromes (e.g., fragile X syndrome, tuberous sclerosis, or Rett syndrome); or (4) current use of psychotropic medication. Common comorbid conditions, such as attention deficit hyperactivity disorder, tic disorders, and emotional disorders, were not grounds for exclusion. During the same period, typically developing (TD) children without any neurological or psychiatric disorders were recruited from local schools to serve as the healthy control group.

**Figure 1:**
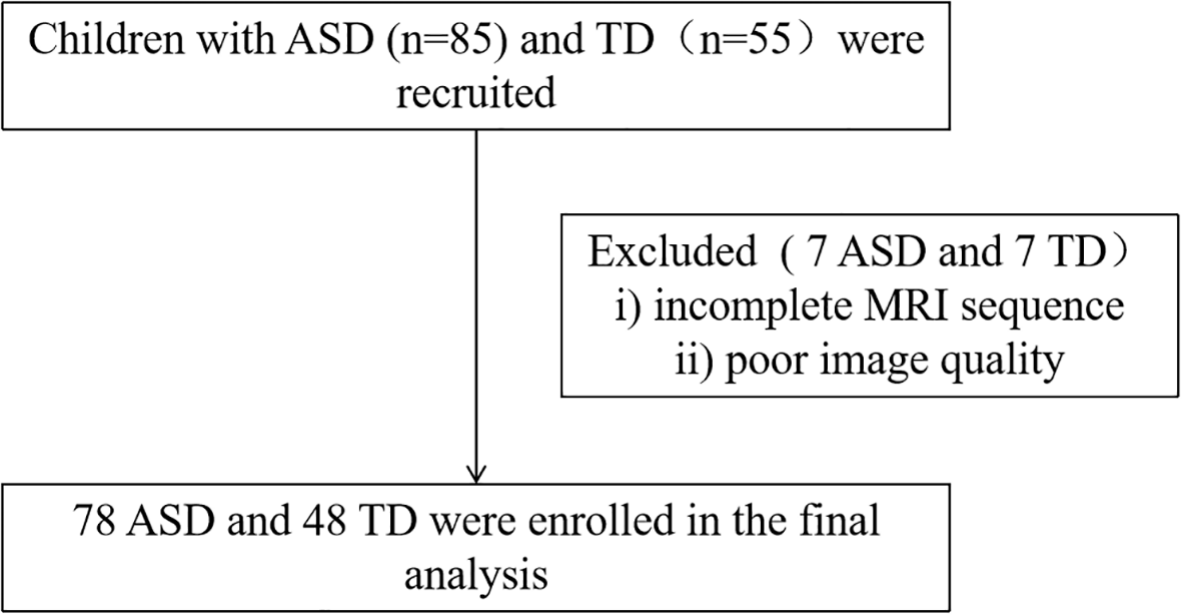
Study flowchart.

After recruitment, ASD diagnosis was confirmed and clinical symptoms were evaluated through a parent interview using the Autism Diagnostic Interview-Revised (ADI_R) and a direct child assessment using the Autism Diagnostic Observation Schedule (ADOS). These assessments were conducted by psychiatrists who had received standardized training. All participants subsequently completed an intelligence quotient (IQ) test and the Beery-Buktenica Developmental Test of Visual-Motor Integration (Beery VMI). The final step involved undergoing magnetic resonance imaging (MRI) scanning. Ultimately, 78 individuals with ASD and 48 TD controls were included in the final analysis. The participant recruitment flowchart is presented in **Figure 1**.

### Clinical assessment

Participants underwent a series of clinical assessments. The ADI_R (23) and the ADOS (24) were administered to confirm the ASD diagnosis and evaluate core symptoms. The ADI_R is a standardized, structured parent interview that assesses symptoms across four domains: social interaction, communication, RRB, and evidence of early developmental abnormalities (25). The ADOS is a standardized, interactive observation assessment used to rate ASD-related symptoms during structured activities. It also comprises four subscales: communication, social interaction, imagination/creativity, and RRB.

Intelligence Quotient (IQ) was assessed using the Chinese Wechsler Intelligence Scale for Children (C-WISC) for participants aged 6–16 years, with a separate version available for children aged 4–6 years (26). VMI function was evaluated with the Beery-Buktenica Developmental Test of Visual-Motor Integration, 6th edition (Beery VMI) (27). This instrument includes three subtests: VMI, Visual Perception (VP), and Motor Coordination (MC), each containing 30 items. Testing was discontinued after three consecutive errors or a 3-minute time limit per subtest, whichever occurred first. Each correct response was awarded one point. The Beery VMI demonstrates high internal consistency (VMI α = 0.92; VP α = 0.91; MC α = 0.90) and provides age-standardized normative scores. It is widely used to assess VMI function in pediatric and adult populations with neurodevelopmental disorders.

### MRI sequences

MRI data were acquired using a 3-Tesla scanner (Philips, Achieva, TX, Best, The Netherlands) equipped with a 32-channel head coil. Diffusion tensor imaging (DTI) was performed with a two-dimensional diffusion-weighted echo-planar imaging sequence using the following parameters: repetition time (TR) = 10,295 ms; echo time (TE) = 91 ms; field of view (FOV) = 128 × 128 mm²; matrix size = 128 × 128; voxel size = 2 × 2 × 2 mm³; 32 diffusion encoding directions; b-value = 1000 s/mm²; 75 interleaved slices with no gap; slice thickness = 2 mm.

### Calculation of the DTI-ALPS index

The DTI-ALPS method was implemented as previously detailed (6), and a schematic summary is provided in **Supplementary Figure S1**. DTI data were processed using the FMRIB Software Library (version 6.0.7.2, http://www.fmrib.ox.ac.uk/fsl/). Preprocessing included correction for off-resonance and eddy current-induced distortions, as well as for subject motion and outliers. Color-coded fractional anisotropy (FA) maps and directional diffusivity maps (x, y, z-axes) were subsequently generated. Based on the FA maps, projection and association fibers were identified. Spherical regions of interest (ROIs) with a 5-mm diameter were manually placed within these bilateral fibers at the level of the lateral ventricle body. Diffusivity values (Dxx, Dyy, Dzz) were extracted from each ROI for ALPS index calculation. The index for each hemisphere was calculated using the formula: $ALPS\;index\;=\;\frac{mean\;(Dxproj,\;Dxassoc)}{mean\;(Dyproj,\;Dzassoc)}$ . The mean ALPS index was then computed as the average of the left and right hemispheric indices.

### Statistical analysis

All statistical analyses were performed using SPSS 26.0 (Statistical Package for Social Sciences, SPSS Inc). The normality of continuous variables was assessed using the Shapiro-Wilk or Kolmogorov-Smirnov test. Group comparisons were conducted as follows: two-sample t-tests for normally distributed continuous variables, Mann-Whitney U tests for non-normally distributed variables, and chi-squared tests for categorical variables. Correlations between the DTI-ALPS index and age, clinical symptoms, and VMI function were examined using Pearson’s correlation (for normal data) or Spearman’s rank correlation (for non-normal data). A mediation analysis was employed to explore the relationships among the DTI-ALPS index, VMI function, and clinical symptoms. Gender, age, and IQ were included as covariates in all correlation and mediation analyses. A p-value< 0.05 was considered statistically significant.

## Results

### Demographic and clinical data

Detailed demographic and clinical characteristics of the ASD and TD groups are presented in **Table 1**. The study enrolled 78 individuals with ASD (72 males, 6 females; mean age 8.17 ± 3.41 years) and 48 TD volunteers (31 males, 17 females; mean age 9.02 ± 2.44 years). No significant difference was found between the groups in terms of age (t = -1.62, p = 0.108). However, the ASD group had a significantly lower IQ than the TD group (t = -5.12, p< 0.001) and a significantly higher proportion of males (χ² = 15.31, p< 0.001). Additionally, the ASD group scored significantly lower on the VP (t = -3.87, p< 0.001), MC (t = -3.67, p< 0.001), and VMI (t = -4.66, p< 0.001) subtests compared to the TD group.

**Table 1:** Demographic and clinical characteristics of the participants.

Variable	ASD (n = 78)	TD (n = 48)	t/χ2	p
Age (years, M ± SD)	8.17 ± 3.41	9.02 ± 2.44	-1.62	0.108
Gender (M/F)	72/6	31/17	15.31	<0.001
IQ (M ± SD)	88.30 ± 19.57	104.40 ± 14.85	-5.12	<0.001
Beery VMI (M ± SD)				
VP	102.55 ± 13.40	112.17 ± 11.88	-3.87	<0.001
MC	98.00 ± 17.97	109.40 ± 13.00	-3.67	<0.001
VMI	94.88 ± 15.08	107.33 ± 11.81	-4.66	<0.001
ADI_R (M ± SD)				
Social interaction	16.38 ± 6.68	–	–	–
Communication	19.55 ± 8.89	–	–	–
RRB	4.13 ± 2.71	–	–	–
Development	2.60 ± 1.78	–	–	–
Total	35.96 ± 13.12	–	–	–
ADOS (M ± SD)				
Communication	5.75 ± 2.35	–	–	–
Social interaction	9.47 ± 2.91	–	–	–
Imagination/creativity	1.55 ± 1.28	–	–	–
RRB	1.61 ± 1.26	–	–	–
Total	18.39 ± 5.64	–	–	–

ASD, autism spectrum disorder; TD, typical developed group; IQ, intelligence quotient; Beery VMI, The Beery-Buktenica Developmental Test of Visual-Motor Integration; VP, visual perception; MC, motor coordination; VMI, visual-motor integration; ADI-R, autism diagnostic interview-revised; RRB, restricted and repetitive behaviors; ADOS, autism diagnostic observation schedule. M, mean; SD, standard deviation; “-” means no available data; M, mean; SD, standard deviation.

### Comparison of the DTI-ALPS index between the two groups

In the ASD group, the DTI-ALPS indices were as follows: 1.49 ± 0.13 for the left hemisphere (DTI-ALPS-L), 1.42 ± 0.13 for the right hemisphere (DTI-ALPS-R), and 1.46 ± 0.11 for the whole-brain average (mean DTI-ALPS). The corresponding values in the TD group were 1.54 ± 0.14, 1.47 ± 0.12, and 1.51 ± 0.11, respectively. As shown in **Table 2**; **Supplementary Figure S2**, all three indices were significantly lower in the ASD group compared to the TD group: DTI-ALPS-L (t = -2.19, p = 0.031), DTI-ALPS-R (t = -2.13, p = 0.035), and mean DTI-ALPS (t = -2.43, p = 0.017).

**Table 2:** Comparison of the DTI-ALPS index between ASD and TD groups.

Variable	ASD (n=78)	TD (n=48)	t	p
DTI-ALPS-L (M ± SD)	1.49 ± 0.13	1.54 ± 0.14	-2.19	0.031
DTI-ALPS-R (M ± SD)	1.42 ± 0.13	1.47 ± 0.12	-2.13	0.035
Mean DTI-ALPS (M ± SD)	1.46 ± 0.11	1.51 ± 0.11	-2.43	0.017

ASD, autism spectrum disorder; TD, typical developed group; DTI-ALPS, Diffusion Tensor Imaging along Perivascular Spaces; Mean DTI-ALPS, average DTI-ALPS index for both left and right hemispheres; L, left-hemispheric; R, right-hemispheric; M, mean; SD, standard deviation.

Furthermore, we examined the effects of group and brain hemisphere on the DTI-ALPS index using a two-way ANOVA. The analysis revealed significant main effects of group (F = 9.304, p = 0.003) and hemisphere (F = 16.675, p< 0.001), but no significant group × hemisphere interaction (F = 0.022, p = 0.883). *Post-hoc* tests indicated that the DTI-ALPS index was significantly higher in the left hemisphere (DTI-ALPS-L) than in the right hemisphere (DTI-ALPS-R) for both the ASD (t = 3.182, p = 0.002) and TD (t = 2.679, p = 0.008) groups (**Supplementary Figure S1**).

Given the known male predominance in ASD, we assessed its effect on the DTI-ALPS index using a two-way ANOVA with group and sex as factors. The analysis revealed no significant main effect of sex. To examine the influence of age, participants were categorized into three developmental stages: preschool (≤6 years), school-age (7–12 years), and adolescence (>12 years). Again, no significant main effect of age was found. Detailed results are presented in **Supplementary Tables S1**, **S2**.

### Correlation analysis in the ASD group

To explore the relationships between the DTI-ALPS indices and age, clinical symptoms, and VMI function in ASD patients, correlation analyses were conducted. The results are presented in **Table 3**. No significant correlations were found between the DTI-ALPS indices and age.

**Table 3:** Correlation analysis in the ASD group.

Variable	DTI-ALPS-L		DTI-ALPS-R		Mean DTI-ALPS	
	r	p	r	p	r	p
Age	0.085	0.472	0.000	0.997	0.048	0.689
IQ	0.072	0.545	0.090	0.450	0.092	0.441
ADI_R						
Social interaction	-0.199	0.087	-0.125	0.284	-0.183	0.117
Communication	-0.355	0.002	-0.319	0.005	-0.381	0.001
RRB	-0.126	0.28	0.039	0.738	-0.048	0.682
Development	-0.126	0.281	-0.179	0.124	-0.173	0.138
Total	-0.287	0.013	-0.218	0.06	-0.285	0.013
ADOS						
Communication	0.042	0.72	0.036	0.759	0.044	0.707
Social interaction	-0.065	0.58	0.164	0.16	0.058	0.624
Imagination/creativity	0.132	0.261	0.056	0.635	0.105	0.368
RRB	-0.095	0.417	-0.242	0.036	-0.192	0.1
Total	-0.009	0.94	0.057	0.63	0.027	0.815
Beery VMI						
VP	0.380	0.004	0.325	0.014	0.402	0.002
MC	0.259	0.052	0.108	0.422	0.209	0.119
VMI	0.385	0.003	0.271	0.041	0.374	0.004

ASD, autism spectrum disorder; DTI-ALPS, Diffusion Tensor Imaging along Perivascular Spaces; Mean DTI-ALPS, average DTI-ALPS index for both left and right hemispheres; L, left-hemispheric; R, right-hemispheric; IQ, intelligence quotient; ADI-R, autism diagnostic interview-revised; RRB, restricted and repetitive behaviors; ADOS, autism diagnostic observation schedule; Beery VMI, The Beery-Buktenica Developmental Test of Visual-Motor Integration; VP, visual perception; MC, motor coordination; VMI, visual-motor integration.

**Figure 2:**
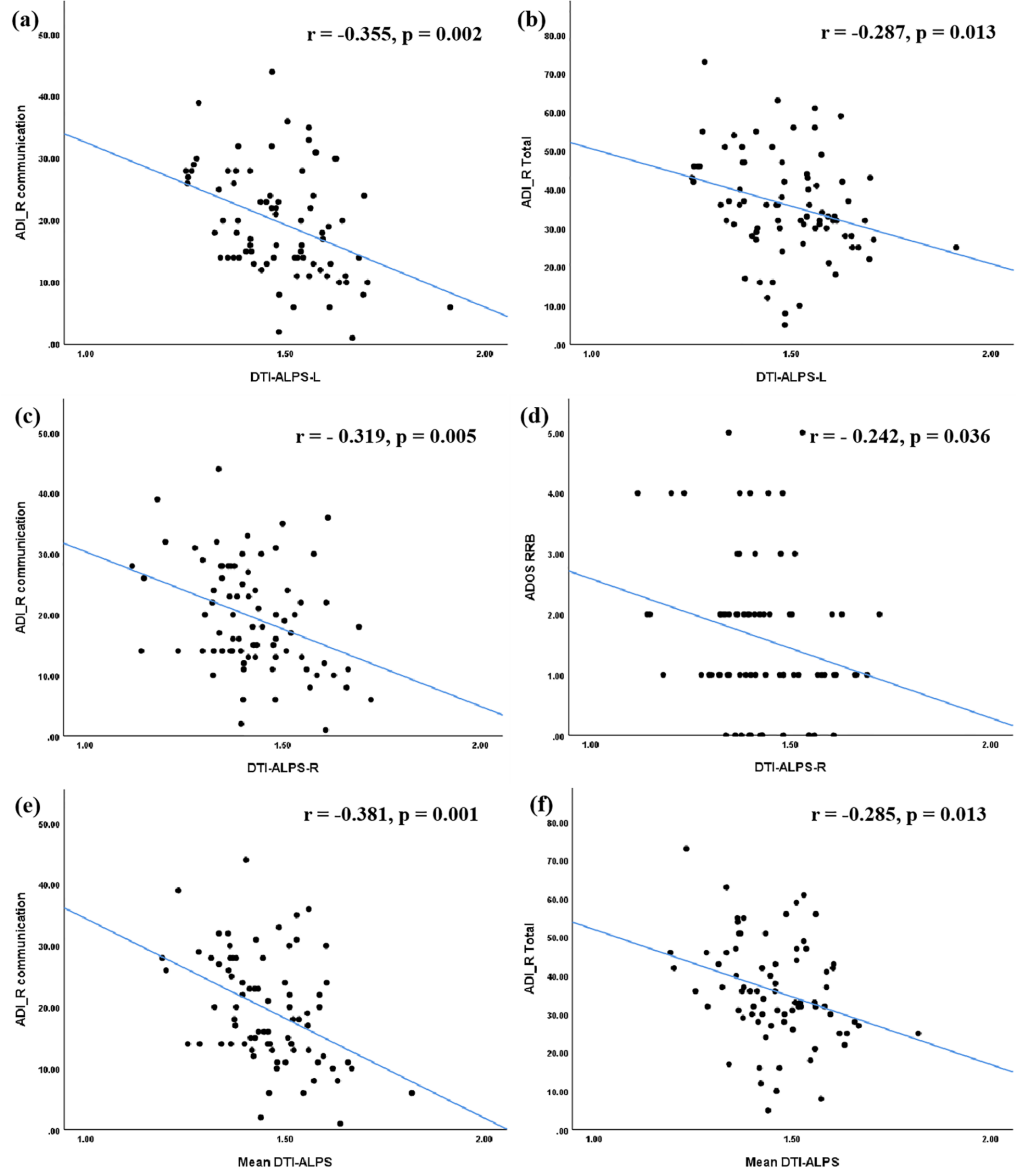
Correlations between the DTI-ALPS index and clinical symptoms in ASD group. The DTI-ALPS-L index **(a, b)** and the Mean DTI-ALPS index **(e, f)** were significantly correlated with ADI_R communication score and total score. The DTI-ALPS-R index was significantly correlated with ADI_R communication score and ADOS RRB score **(c, d)**. ASD, autism spectrum disorder; TD, typical developed group; DTI-ALPS, Diffusion Tensor Imaging along Perivascular Spaces; Mean DTI-ALPS, average DTI-ALPS index for both left and right hemispheres; L, left-hemispheric; R, right-hemispheric. ADI_R, autism diagnostic interview-revised; ADOS, autism diagnostic observation schedule; RRB, restricted and repetitive behaviors.

Regarding clinical symptoms, significant negative correlations were observed between ADI_R Communication score and the DTI-ALPS-L (r = -0.355, p = 0.002), DTI-ALPS-R (r = -0.319, p = 0.005), and mean DTI-ALPS (r = -0.381, p = 0.001). Additionally, both DTI-ALPS-L (r = -0.287, p = 0.013) and the mean DTI-ALPS (r = -0.285, p = 0.013) were negatively correlated with the ADI_R total score. A significant negative correlation was also found between DTI-ALPS-R and the ADOS RRB score (r = -0.242, p = 0.036) (**Figure 2**).

**Figure 3:**
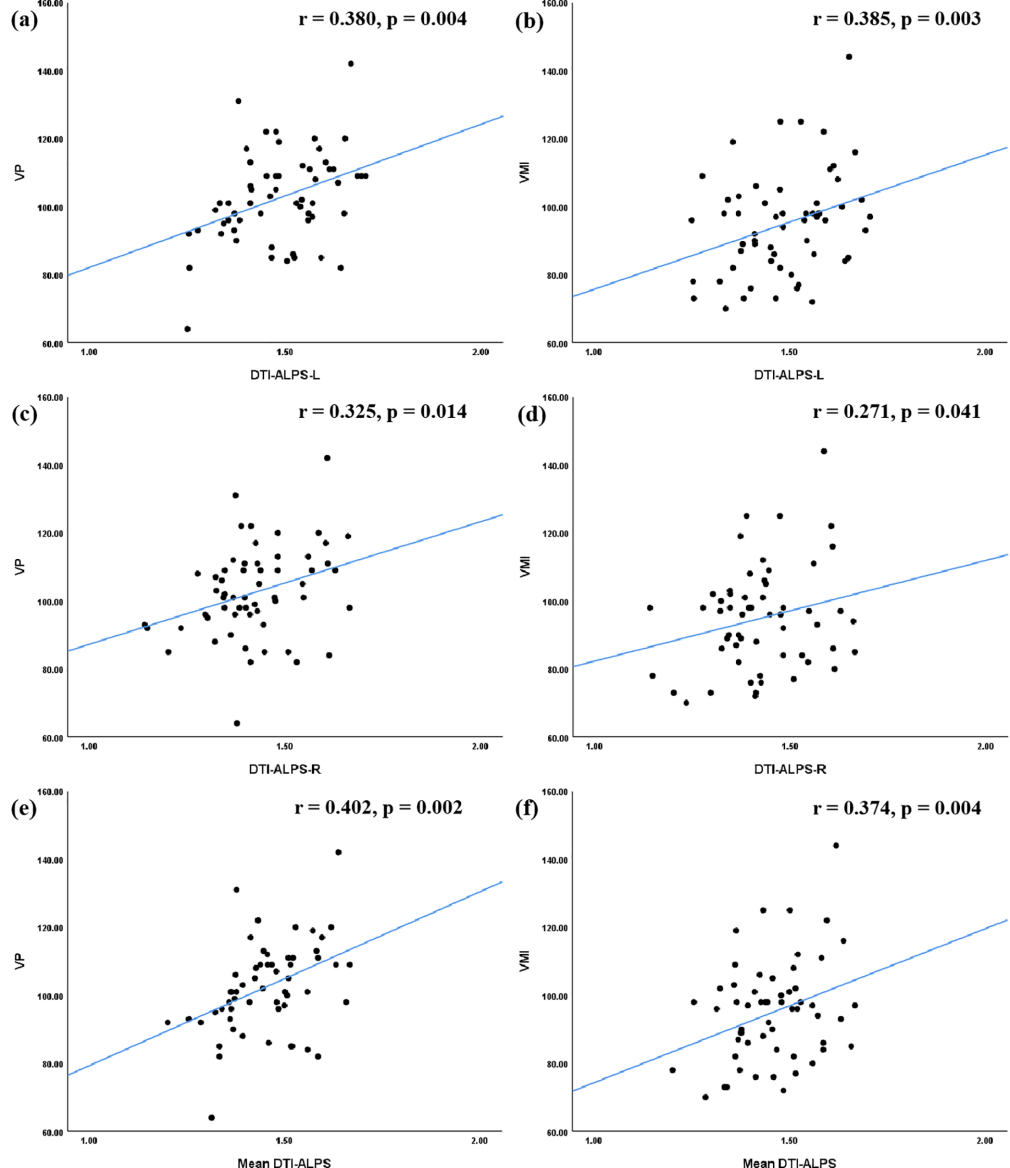
Correlations between the DTI-ALPS index and VMI function in ASD group. In terms of the relationship between the DTI-ALPS index and VMI function, the results of correlation analyses indicated that the DTI-ALPS-L index **(a, b)**, the DTI-ALPS-R index**(c, d)**, and the Mean DTI-ALPS **(e, f)** were significantly correlated with VP score, as well as with VMI score. ASD, autism spectrum disorder; TD, typical developed group; DTI-ALPS, Diffusion Tensor Imaging along Perivascular Spaces; Mean DTI-ALPS, average DTI-ALPS index for both left and right hemispheres; L, left-hemispheric; R, right-hemispheric; VP, visual perception; MC, motor coordination; VMI, visual-motor integration.

In terms of VMI function, significant positive correlations were identified. The DTI-ALPS-L (r = 0.380, p = 0.004), DTI-ALPS-R (r = -0.325, p = 0.014), and mean DTI-ALPS (r = 0.402, p = 0.002) were all positively correlated with the VP score. Similarly, these indices were positively correlated with the VMI score (DTI-ALPS-L: r = 0.385, p = 0.003; DTI-ALPS-R: r = 0.271, p = 0.041; mean DTI-ALPS: r = 0.374, p = 0.004) (**Figure 3**).

### Mediation analysis in the ASD group

**Figure 4:**
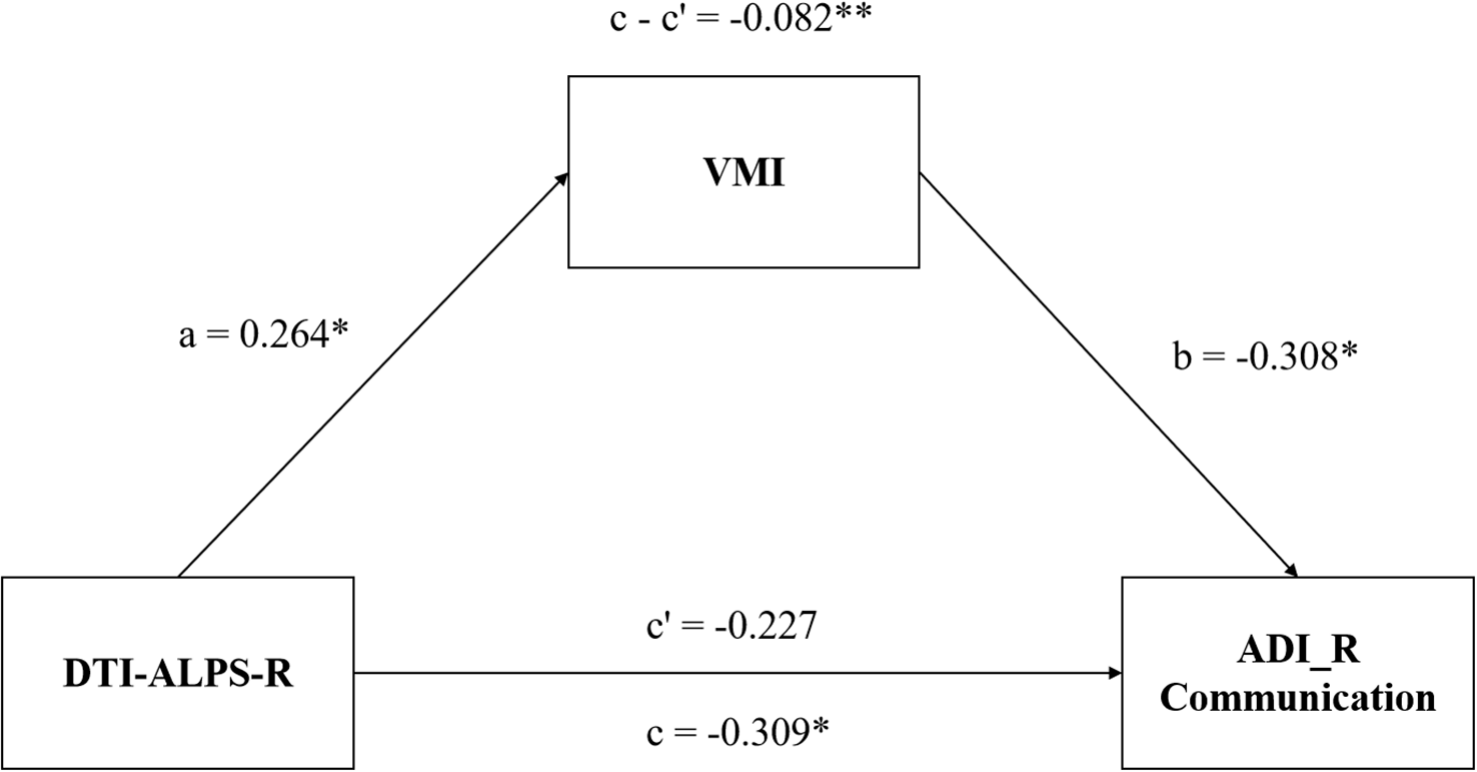
Mediation model of DTI-ALPS-R, VMI function and symptoms of children with ASD. The VMI function mediates the relationship between DTI-ALPS-R and the communication deficiency of ASD. ASD, autism spectrum disorder; DTI-ALPS, Diffusion Tensor Imaging along Perivascular Spaces; R, right-hemispheric; VMI, visual-motor integration; ADI_R communication, communication subscale of the autism diagnostic interview-revised (ADI_R).*, P< 0.05; **, P< 0.01.

We conducted a mediation analysis to elucidate the relationships among the DTI-ALPS index, VMI function, and clinical symptoms. In the model, the DTI-ALPS index was the independent variable, VMI function was the mediator, and clinical symptoms were the outcome variables, with adjustments for age, gender, and IQ. The analysis revealed a significant mediating effect of VMI score on the relationship between DTI-ALPS-R and the ADI_R Communication score (indirect effect β = -0.082, p< 0.001; **Figure 4**).

## Discussion

This study utilized non-invasive MRI techniques to investigate glymphatic system function in children with ASD compared to TD controls. Our principal findings were: (i) the DTI-ALPS index was significantly reduced in the ASD group; (ii) the DTI-ALPS index correlated negatively with scores on the ADI_R Communication and Total scales, as well as the ADOS RRB scale, but positively with VP and VMI scores; and (iii) VMI scores mediated the relationship between the DTI-ALPS-R and ADI_R Communication scores. These results provide compelling evidence for glymphatic dysfunction in ASD, reinforcing its potential role in the disorder’s pathophysiology.

The significantly lower DTI-ALPS index observed in children with ASD indicates compromised glymphatic function and a diminished capacity for cerebral waste clearance. Perivascular spaces (PVS), which are integral components of the glymphatic system and serve as primary conduits for waste transport, are known to cause glymphatic stasis when dilated. Previous neuroimaging studies have consistently reported an increased prevalence of enlarged PVS in ASD (28– 29), providing a plausible anatomical basis for the glymphatic dysfunction observed here. Furthermore, the ASD brain exhibits robust neuroinflammation, evidenced by microglial and astrocytic activation (30) and elevated levels of pro-inflammatory cytokines such as TNF-α, IL-6, GM-CSF, and IFN-γ (31). PET studies have further confirmed significant microglial activation across multiple brain regions in ASD (32). Reactive astrogliosis contributes to this process by generating inflammatory mediators, inducing glial morphological changes, and promoting immune cell accumulation within PVS, collectively impairing clearance (33, 34). In line with Li et al. (13), our study provides direct neuroimaging evidence of compromised glymphatic waste clearance in ASD.

We also found that the DTI-ALPS index correlated inversely with the severity of core ASD symptoms—specifically, communication deficits and RRB. This suggests that poorer cerebral waste clearance (indicated by a lower ALPS index) is associated with greater clinical severity. Impaired glymphatic function reduces the clearance of inflammatory mediators (35), leading to their accumulation in PVS. These accumulated factors perpetuate microglial and astrocytic activation, engage NF-κB signaling, and trigger further release of pro-inflammatory cytokines and chemokines, thereby amplifying and chronicifying neuroinflammation (36, 37). This creates a self-reinforcing cycle wherein impaired clearance and sustained neuroinflammation disrupt cerebral microstructure and function, ultimately contributing to ASD-related behavioral alterations (38). Additionally, reduced clearance of misfolded proteins such as APP, Aβ, and tau has been implicated in the aberrant brain changes observed in ASD (39, 40). Ray et al. (41), for example, reported dysregulated levels of soluble APP fragments (sAPPα and sAPPβ) in individuals with ASD, which correlated with clinical phenotypes. Collectively, these findings position diminished waste clearance as a potential pathogenic contributor to the neurobiology of ASD.

Furthermore, we found that VMI function was significantly impaired in children with ASD and was positively correlated with the DTI-ALPS index. Mediation analysis indicated that VMI mediates the relationship between DTI-ALPS-R and communication deficits, suggesting a pathway through which glymphatic impairment contributes to communicative impairment. VMI deficits are linked to learning difficulties involving language and symbols processing (42, 43) and may underlie specific language impairment (44). Neuroimaging studies suggest that VMI and language functions share neural substrates, including the cerebellum—a region implicated in both motor coordination and language processing in ASD (45, 46). As a core component of perception-action coupling (47), VMI disruption may impair the translation of social cues (e.g., prosody, gesture) into appropriate motor and communicative responses, a key deficit in ASD (48, 49). Thus, our findings suggest that glymphatic dysfunction may contribute to communication deficits in ASD via disrupted visuomotor integration, although the precise mechanisms require further investigation.

An intriguing finding of our study was the lack of a significant correlation between the DTI-ALPS index and age in the ASD group. While this appears to be at odds with reports of transiently elevated extra-axial CSF (EA-CSF) volume in early infancy (50–53), it may instead reflect distinct pathophysiological trajectories at macroscopic and microscopic levels of CSF dynamics. The normalization of EA-CSF likely results from the delayed maturation of macroscopic drainage structures, such as the meningeal lymphatic vessels (54–56). In contrast, glymphatic activity, as measured by the DTI-ALPS index, depends on the microstructural integrity of PVS and the polarized expression of Aquaporin 4 (AQP4) channels in astrocytic endfeet. This microscopic function may sustain a more lasting impairment due to early circulatory disturbances or neuroinflammation (57–59). Critically, recent evidence provides a pivotal link, demonstrating that enlarged perivascular spaces are most prominent in younger children with ASD and may be sequalae of early EA-CSF excess (50). This suggests that early macroscopic abnormalities may lead to long-term alterations at the microscopic level. Consequently, the observed “decoupling” between the DTI-ALPS index and age in our study strongly indicates that glymphatic dysfunction in ASD may represent an early-onset and persistent endophenotype, rather than a transient developmental phase. This positions the DTI-ALPS index as a potential and reliable biomarker for investigating the enduring neuropathological mechanisms of ASD beyond early childhood. We acknowledge that this interpretation requires confirmation through future longitudinal studies.

This study has several limitations. First, its cross-sectional nature precludes causal inferences regarding the relationship between glymphatic dysfunction and ASD symptoms. Longitudinal studies are needed to determine whether glymphatic alterations precede or result from ASD. Second, although comparable in size to many neuroimaging studies of ASD, our sample may limit the generalizability of the findings. Finally, the DTI-ALPS index is derived from diffusion measures around PVSs at the level of the lateral ventricles in a single slice, which may not capture regional variations in glymphatic clearance throughout the brain.

## Conclusion

Using the DTI-ALPS method, we demonstrated significantly reduced glymphatic function in children with ASD, which correlated with the severity of core symptoms. These findings implicate impaired glymphatic clearance in ASD pathogenesis. Furthermore, mediation analysis suggested that deficits in visuomotor integration link glymphatic dysfunction to communication impairments. Collectively, our study provides neuroimaging evidence for the role of the glymphatic system in ASD and identifies potential biomarkers for future mechanistic and interventional research.

## Data Availability

The raw data supporting the conclusions of this article will be made available by the authors, without undue reservation.

## References

[1] American Psychiatric Association. Diagnostic and statistical manual of mental disorders: DSM-5™. 5th ed. Arlington, VA, US: American Psychiatric Publishing, Inc (2013). 947 p. doi: 10.1176/appi.books.9780890425596

[2] Fuentes J Hervás A Howlin P ESCAP ASD Working Party. ESCAP practice guidance for autism: a summary of evidence-based recommendations for diagnosis and treatment. Eur Child Adolesc Psychiatry. (2021) 30:961–84. doi: 10.1007/s00787-020-01587-4, PMID: 32666205 PMC8140956

[3] Mestre H Mori Y Nedergaard M. The brain’s glymphatic system: current controversies. Trends Neurosci. (2020) 43:458–66. doi: 10.1016/j.tins.2020.04.003, PMID: 32423764 PMC7331945

[4] Rasmussen MK Mestre H Nedergaard M. Fluid transport in the brain. Physiol Rev. (2022) 102:1025–151. doi: 10.1152/physrev.00031.2020, PMID: 33949874 PMC8897154

[5] van der Thiel MM Backes WH Ramakers IHGB Jansen JFA. Novel developments in non-contrast enhanced MRI of the perivascular clearance system: What are the possibilities for Alzheimer’s disease research? Neurosci Biobehav Rev. (2023) 144:104999. doi: 10.1016/j.neubiorev.2022.104999, PMID: 36529311

[6] Taoka T Masutani Y Kawai H Nakane T Matsuoka K Yasuno F. Evaluation of glymphatic system activity with the diffusion MR technique: diffusion tensor image analysis along the perivascular space (DTI-ALPS) in Alzheimer’s disease cases. Jpn J Radiol. (2017) 35:172–8. doi: 10.1007/s11604-017-0617-z, PMID: 28197821

[7] Hsu J-L Wei Y-C Toh CH Hsiao I-T Lin K-J Yen T-C. Magnetic resonance images implicate that glymphatic alterations mediate cognitive dysfunction in alzheimer disease. Ann Neurol. (2023) 93:164–74. doi: 10.1002/ana.26516, PMID: 36214568 PMC10091747

[8] Meng J-C Shen M-Q Lu Y-L Feng H-X Chen X-Y Xu D-Q. Correlation of glymphatic system abnormalities with Parkinson’s disease progression: a clinical study based on non-invasive fMRI. J Neurol. (2024) 271:457–71. doi: 10.1007/s00415-023-12004-6, PMID: 37755462

[9] Bah TM Siler DA Ibrahim AH Cetas JS Alkayed NJ. Fluid dynamics in aging-related dementias. Neurobiol Dis. (2023) 177:105986. doi: 10.1016/j.nbd.2022.105986, PMID: 36603747 PMC12369312

[10] Yang C Tian S Du W Liu M Hu R Gao B. Glymphatic function assessment with diffusion tensor imaging along the perivascular space in patients with major depressive disorder and its relation to cerebral white-matter alteration. Quant Imaging Med Surg. (2024) 14:6397–412. doi: 10.21037/qims-24-510, PMID: 39281139 PMC11400689

[11] Abdolizadeh A Torres-Carmona E Kambari Y Amaev A Song J Ueno F. Evaluation of the glymphatic system in schizophrenia spectrum disorder using proton magnetic resonance spectroscopy measurement of brain macromolecule and diffusion tensor image analysis along the perivascular space index. Schizophr Bull. (2024) 50:1396–410. doi: 10.1093/schbul/sbae060, PMID: 38748498 PMC11548937

[12] Tu Y Fang Y Li G Xiong F Gao F. Glymphatic system dysfunction underlying schizophrenia is associated with cognitive impairment. Schizophr Bull. (2024) 50:1223–31. doi: 10.1093/schbul/sbae039, PMID: 38581275 PMC11349007

[13] Li X Ruan C Zibrila AI Musa M Wu Y Zhang Z. Children with autism spectrum disorder present glymphatic system dysfunction evidenced by diffusion tensor imaging along the perivascular space. Med (Baltimore). (2022) 101:e32061. doi: 10.1097/MD.0000000000032061, PMID: 36482590 PMC9726346

[14] Neely KA Mohanty S Schmitt LM Wang Z Sweeney JA Mosconi MW. Motor memory deficits contribute to motor impairments in autism spectrum disorder. J Autism Dev Disord. (2019) 49:2675–84. doi: 10.1007/s10803-016-2806-5, PMID: 27155985 PMC5099114

[15] Nardone R Langthaler PB Schwenker K Kunz AB Sebastianelli L Saltuari L. Visuomotor integration in early Alzheimer’s disease: A TMS study. J Neurol Sci. (2022) 434:120129. doi: 10.1016/j.jns.2021.120129, PMID: 34998240

[16] Zampella CJ Wang LAL Haley M Hutchinson AG de Marchena A. Motor skill differences in autism spectrum disorder: a clinically focused review. Curr Psychiatry Rep. (2021) 23:64. doi: 10.1007/s11920-021-01280-6, PMID: 34387753

[17] Widmer FC O’Toole SM Keller GB. NMDA receptors in visual cortex are necessary for normal visuomotor integration and skill learning. Elife. (2022) 11:e71476. doi: 10.7554/eLife.71476, PMID: 35170429 PMC8901170

[18] Hume K Steinbrenner JR Odom SL Morin KL Nowell SW Tomaszewski B. Evidence-based practices for children, youth, and young adults with autism: third generation review. J Autism Dev Disord. (2021) 51:4013–32. doi: 10.1007/s10803-020-04844-2, PMID: 33449225 PMC8510990

[19] Deng J Lei T Du X. Effects of sensory integration training on balance function and executive function in children with autism spectrum disorder: evidence from Footscan and fNIRS. Front Psychol. (2023) 14:1269462. doi: 10.3389/fpsyg.2023.1269462, PMID: 37946875 PMC10631781

[20] Hayes MT. Parkinson’s disease and parkinsonism. Am J Med. (2019) 132:802–7. doi: 10.1016/j.amjmed.2019.03.001, PMID: 30890425

[21] Shen T Yue Y Ba F He T Tang X Hu X. Diffusion along perivascular spaces as marker for impairment of glymphatic system in Parkinson’s disease. NPJ Parkinsons Dis. (2022) 8:1–10. doi: 10.1038/s41531-022-00437-1, PMID: 36543809 PMC9772196

[22] He P Shi L Li Y Duan Q Qiu Y Feng S. The association of the glymphatic function with parkinson’s disease symptoms: neuroimaging evidence from longitudinal and cross-sectional studies. Ann Neurol. (2023) 94:672–83. doi: 10.1002/ana.26729, PMID: 37377170

[23] Lord C Rutter M Le Couteur A. Autism Diagnostic Interview-Revised: a revised version of a diagnostic interview for caregivers of individuals with possible pervasive developmental disorders. J Autism Dev Disord. (1994) 24:659–85. doi: 10.1007/BF02172145, PMID: 7814313

[24] Lord C Risi S Lambrecht L Cook EH Leventhal BL DiLavore PC. The autism diagnostic observation schedule-generic: a standard measure of social and communication deficits associated with the spectrum of autism. J Autism Dev Disord. (2000) 30:205–23. doi: 10.1023/A:1005592401947, PMID: 11055457

[25] Saemundsen E Magnússon P Smári J Sigurdardóttir S. Autism diagnostic interview-revised and the childhood autism rating scale: convergence and discrepancy in diagnosing autism. J Autism Dev Disord. (2003) 33:319–28. doi: 10.1023/A:1024410702242, PMID: 12908834

[26] Li D Jin Y Vandenberg SG Zhu YM Tang CH. Report on Shanghai norms for the Chinese translation of the Wechsler Intelligence Scale for Children-Revised. Psychol Rep. (1990) 67:531–41. doi: 10.2466/pr0.1990.67.2.531, PMID: 2263706

[27] Beery-Buktenica Developmental Test of Visual-Motor Integration. The (BEERY™ VMI) – Pearson Clinical. Available online at: https://pearsonclinical.in/solutions/beery-buktenica-developmental-test-of-visual-motor-integration-sixth-edition-the-beery-vmi/ (Accessed March 12, 2025).

[28] Boddaert N Zilbovicius M Philipe A Robel L Bourgeois M Barthélemy C. MRI findings in 77 children with non-syndromic autistic disorder. PLoS One. (2009) 4:e4415. doi: 10.1371/journal.pone.0004415, PMID: 19204795 PMC2635956

[29] Taber KH Shaw JB Loveland KA Pearson DA Lane DM Hayman LA. Accentuated Virchow-Robin spaces in the centrum semiovale in children with autistic disorder. J Comput Assist Tomogr. (2004) 28:263–8. doi: 10.1097/00004728-200403000-00017, PMID: 15091132

[30] Morgan JT Chana G Pardo CA Achim C Semendeferi K Buckwalter J. Microglial activation and increased microglial density observed in the dorsolateral prefrontal cortex in autism. Biol Psychiatry. (2010) 68:368–76. doi: 10.1016/j.biopsych.2010.05.024, PMID: 20674603

[31] Li X Chauhan A Sheikh AM Patil S Chauhan V Li X-M. Elevated immune response in the brain of autistic patients. J Neuroimmunol. (2009) 207:111–6. doi: 10.1016/j.jneuroim.2008.12.002, PMID: 19157572 PMC2770268

[32] Suzuki K Sugihara G Ouchi Y Nakamura K Futatsubashi M Takebayashi K. Microglial activation in young adults with autism spectrum disorder. JAMA Psychiatry. (2013) 70:49–58. doi: 10.1001/jamapsychiatry.2013.272, PMID: 23404112

[33] Rustenhoven J Drieu A Mamuladze T de Lima KA Dykstra T Wall M. Functional characterization of the dural sinuses as a neuroimmune interface. Cell. (2021) 184:1000–1016.e27. doi: 10.1016/j.cell.2020.12.040, PMID: 33508229 PMC8487654

[34] Xu J-Q Liu Q-Q Huang S-Y Duan C-Y Lu H-B Cao Y. The lymphatic system: a therapeutic target for central nervous system disorders. Neural Regener Res. (2023) 18:1249–56. doi: 10.4103/1673-5374.355741, PMID: 36453401 PMC9838139

[35] Hsu S-J Zhang C Jeong J Lee S-I McConnell M Utsumi T. Enhanced meningeal lymphatic drainage ameliorates neuroinflammation and hepatic encephalopathy in cirrhotic rats. Gastroenterology. (2021) 160:1315–1329.e13. doi: 10.1053/j.gastro.2020.11.036, PMID: 33227282 PMC7956141

[36] Zhao J Bi W Xiao S Lan X Cheng X Zhang J. Neuroinflammation induced by lipopolysaccharide causes cognitive impairment in mice. Sci Rep. (2019) 9:5790. doi: 10.1038/s41598-019-42286-8, PMID: 30962497 PMC6453933

[37] Gu S Li Y Jiang Y Huang JH Wang F. Glymphatic dysfunction induced oxidative stress and neuro-inflammation in major depression disorders. Antioxidants (Basel). (2022) 11:2296. doi: 10.3390/antiox11112296, PMID: 36421482 PMC9687220

[38] Bjorklund G Saad K Chirumbolo S Kern JK Geier DA Geier MR. Immune dysfunction and neuroinflammation in autism spectrum disorder. Acta Neurobiol Exp (Wars). (2016) 76:257–68. doi: 10.21307/ane-2017-025, PMID: 28094817

[39] Sokol DK Lahiri DK. Neurodevelopmental disorders and microcephaly: how apoptosis, the cell cycle, tau and amyloid-β precursor protein APPly. Front Mol Neurosci. (2023) 16:1201723. doi: 10.3389/fnmol.2023.1201723, PMID: 37808474 PMC10556256

[40] Jęśko H Cieślik M Gromadzka G Adamczyk A. Dysfunctional proteins in neuropsychiatric disorders: From neurodegeneration to autism spectrum disorders. Neurochem Int. (2020) 141:104853. doi: 10.1016/j.neuint.2020.104853, PMID: 32980494

[41] Ray B Long JM Sokol DK Lahiri DK. Increased secreted amyloid precursor protein-α (sAPPα) in severe autism: proposal of a specific, anabolic pathway and putative biomarker. PLoS One. (2011) 6:e20405. doi: 10.1371/journal.pone.0020405, PMID: 21731612 PMC3120811

[42] Brusilovskiy E. Developmental test of visual-motor integration: Administration and scoring manual (1967). Available online at: https://www.academia.edu/60482812/Developmental_test_of_visual_motor_integration_Administration_and_scoring_manual (Accessed April 17, 2025).

[43] Relationship between visuomotor and handwriting skills of children in kindergarten. (Accessed April 17, 2025). 10.5014/ajot.48.11.982 7840134

[44] Nicola K Watter P. Visual–motor integration performance in children with severe specific language impairment. Child. (2016) 42:742–9. doi: 10.1111/cch.12365, PMID: 27291941

[45] Reindal L Nærland T Sund AM Glimsdal BA Andreassen OA Weidle B. The co-occurrence of motor and language impairments in children evaluated for autism spectrum disorder. explorative study Norway. Res Dev Disabil. (2022) 127:104256. doi: 10.1016/j.ridd.2022.104256, PMID: 35580394

[46] Frosch IR Mittal VA D’Mello AM. Cerebellar contributions to social cognition in ASD: A predictive processing framework. Front Integr Neurosci. (2022) 16:810425. doi: 10.3389/fnint.2022.810425, PMID: 35153691 PMC8832100

[47] Whyatt C Craig C. Sensory-motor problems in autism. Front Integr Neurosci. (2013) 7:51. doi: 10.3389/fnint.2013.00051, PMID: 23882194 PMC3714545

[48] Wang LAL Petrulla V Zampella CJ Waller R Schultz RT. Gross motor impairment and its relation to social skills in autism spectrum disorder: A systematic review and two meta-analyses. Psychol Bull. (2022) 148:273–300. doi: 10.1037/bul0000358, PMID: 35511567 PMC9894569

[49] Zampella CJ Bennetto L Herrington JD. Computer vision analysis of reduced interpersonal affect coordination in youth with autism spectrum disorder. Autism Res. (2020) 13:2133–42. doi: 10.1002/aur.2334, PMID: 32666690 PMC7748996

[50] Sotgiu MA Lo Jacono A Barisano G Saderi L Cavassa V Montella A. Brain perivascular spaces and autism: clinical and pathogenic implications from an innovative volumetric MRI study. Front Neurosci. (2023) 17:1205489. doi: 10.3389/fnins.2023.1205489, PMID: 37425010 PMC10328421

[51] Shen MD Nordahl CW Li DD Lee A Angkustsiri K Emerson RW. Extra-axial cerebrospinal fluid in high-risk and normal-risk children with autism aged 2–4 years: a case-control study. Lancet Psychiatry. (2018) 5:895–904. doi: 10.1016/S2215-0366(18)30294-3, PMID: 30270033 PMC6223655

[52] Shen MD Kim SH McKinstry RC Gu H Hazlett HC Nordahl CW. Increased extra-axial cerebrospinal fluid in high-risk infants who later develop autism. Biol Psychiatry. (2017) 82:186–93. doi: 10.1016/j.biopsych.2017.02.1095, PMID: 28392081 PMC5531051

[53] Garic D McKinstry RC Rutsohn J Slomowitz R Wolff J MacIntyre LC. Enlarged perivascular spaces in infancy and autism diagnosis, cerebrospinal fluid volume, and later sleep problems. JAMA Netw Open. (2023) 6:e2348341. doi: 10.1001/jamanetworkopen.2023.48341, PMID: 38113043 PMC10731509

[54] Xu H Lehtinen MK. Cerebrospinal fluid magnetic resonance imaging: improving early diagnosis of autism and other neurodevelopmental conditions. Biol Psychiatry Cognit Neurosci Neuroimaging. (2020) 5:635–7. doi: 10.1016/j.bpsc.2020.05.007, PMID: 32646616

[55] Shen MD Piven J. Brain and behavior development in autism from birth through infancy. Dialogues Clin Neurosci. (2017) 19:325–33. doi: 10.31887/DCNS.2017.19.4/mshen, PMID: 29398928 PMC5789210

[56] Kapoor KG Katz SE Grzybowski DM Lubow M. Cerebrospinal fluid outflow: an evolving perspective. Brain Res Bull. (2008) 77:327–34. doi: 10.1016/j.brainresbull.2008.08.009, PMID: 18793703

[57] Iliff JJ Wang M Liao Y Plogg BA Peng W Gundersen GA. A paravascular pathway facilitates CSF flow through the brain parenchyma and the clearance of interstitial solutes, including amyloid β. Sci Transl Med. (2012) 4:147ra111. doi: 10.1126/scitranslmed.3003748, PMID: 22896675 PMC3551275

[58] Rasmussen MK Mestre H Nedergaard M. The glymphatic pathway in neurological disorders. Lancet Neurol. (2018) 17:1016–24. doi: 10.1016/S1474-4422(18)30318-1, PMID: 30353860 PMC6261373

[59] Sotgiu S Manca S Gagliano A Minutolo A Melis MC Pisuttu G. Immune regulation of neurodevelopment at the mother-foetus interface: the case of autism. Clin Transl Immunol. (2020) 9:e1211. doi: 10.1002/cti2.1211, PMID: 33209302 PMC7662086

