# Supplementary material for: Glymphatic system dysfunction in children with autism spectrum disorder as evidenced by the diffusion tensor imaging along perivascular spaces index
Source: Front Psychiatry. 2025 Oct 29;16:1701816. doi: 10.3389/fpsyt.2025.1701816 (PMC12605367; doi:10.3389/fpsyt.2025.1701816)
Supplement: Supplementary file 1 [file DataSheet1.docx]

**The supplementary materials**

**Table S1 The effects of the group and gender on the DTI-ALPS index**

|  |  | ASD (M±SD) | | TD (M±SD) | | F | p |
| --- | --- | --- | --- | --- | --- | --- | --- |
|  |  | male（n=72） | female（n=6） | male（n=31） | female（n=17） |  |  |
| group*gender | DTI-ALPS-L | 1.49±0.13 | 1.54±0.13 | 1.54±0.14 | 1.54±0.14 | 0.794^a^  0.414^b^  0.661^c^ | 0.375  0.521  0.418 |
|  | DTI-ALPS-R | 1.42±0.13 | 1.47±0.13 | 1.47±0.12 | 1.47±0.12 | 0.614^a^  0.506^b^  0.806^c^ | 0.435  0.478  0.371 |
|  | Mean DTI-ALPS | 1.45±0.11 | 1.50±0.13 | 1.51±0.11 | 1.50±0.12 | 0.892^a^  0.579^b^  0.924^c^ | 0.347  0.448  0.338 |

Note: ASD, autism spectrum disorder; TD, typical developed group; DTI-ALPS, Diffusion Tensor Imaging along Perivascular Spaces; Mean DTI-ALPS, average DTI-ALPS index for both left and right hemispheres; L, left-hemispheric; R, right-hemispheric. ^a^, the main effects of ‘group’; ^b^, the main effects of ‘gender’; ^c^, interaction between ‘group’ and ‘gender’ . M, mean; SD, standard deviation.

**Table S2 The effects of the group and age on the DTI-ALPS index**

|  |  | ASD (M±SD) | | | TD (M±SD) | | | F | p |
| --- | --- | --- | --- | --- | --- | --- | --- | --- | --- |
|  |  | ≤6 years  (n=31） | 6~12 years（n=37） | ＞12 years（n=10） | ≤6 years（n=5） | 6~12 years（n=39） | ＞12 years（n=4） |  |  |
| group*age | DTI-ALPS-L | 1.47±0.14 | 1.49±0.10 | 1.56±0.18 | 1.51±0.16 | 1.55±0.13 | 1.53±0.18 | 0.510^a^  0.521^b^  0.533^c^ | 0.468  0.595  0.588 |
|  | DTI-ALPS-R | 1.43±0.13 | 1.40±0.12 | 1.49±0.14 | 1.39±0.05 | 1.49±0.12 | 1.44±0.16 | 0.000^a^  0.759^b^  2.557^c^ | 0.999  0.471  0.082 |
|  | Mean DTI-ALPS | 1.45±0.11 | 1.45±0.10 | 1.52±0.16 | 1.45±0.10 | 1.52±0.11 | 1.48±0.16 | 0.182^a^  0.784^b^  1.527^c^ | 0.67  0.459  0.221 |

Note: ASD, autism spectrum disorder; TD, typical developed group; DTI-ALPS, Diffusion Tensor Imaging along Perivascular Spaces; Mean DTI-ALPS, average DTI-ALPS index for both left and right hemispheres; L, left-hemispheric; R, right-hemispheric. ^a^, the main effects of ‘group’; ^b^, the main effects of ‘age’; ^c^, interaction between ‘group’ and ‘age’ . M, mean; SD, standard deviation.


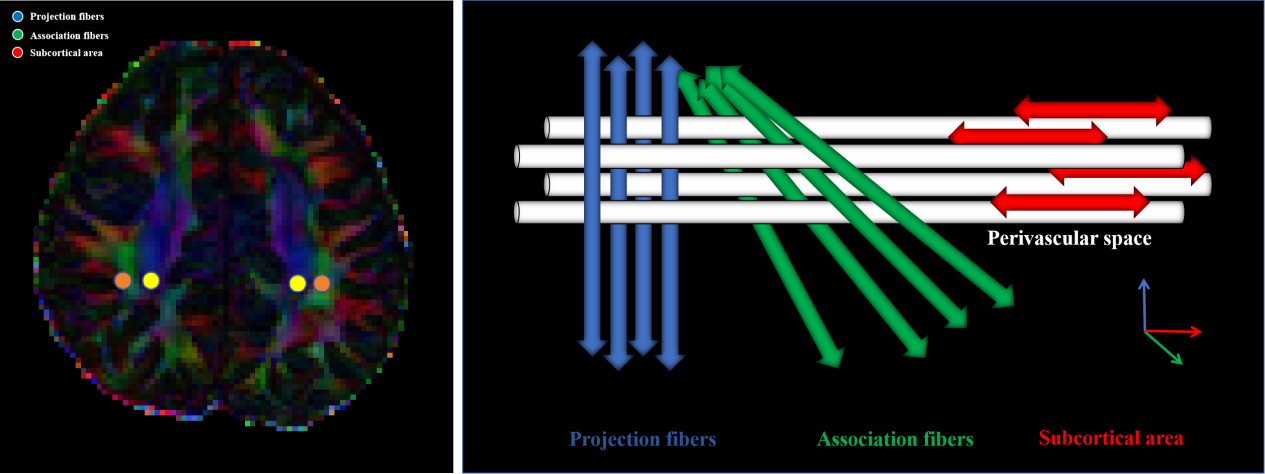


**Fig S1 The concept and results of the difusion tensor mage-analysis along with the perivascular space (DTI-ALPS) method for perivascular difusion.**


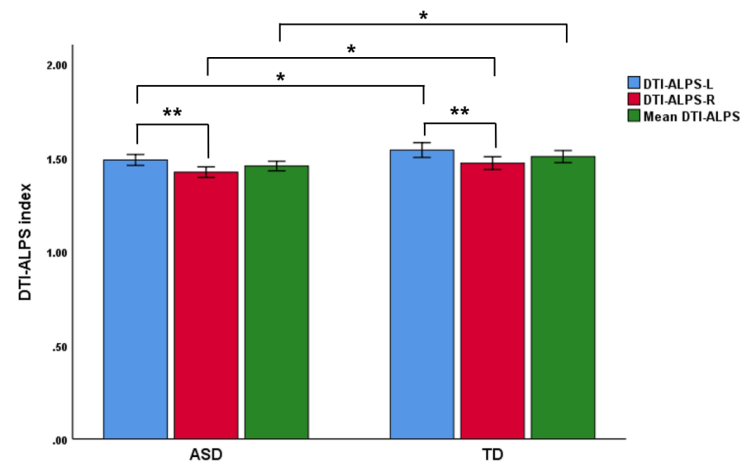


**Fig S2** **DTI-ALPS index variations: inter- and intra-group analysis.** There are significant differences in the DTI-ALPS index between the left and right sides within the ASD and TD groups. In addition, DTI-ALPS-L, DTI-ALPS-R, Mean DTI-ALPS in the ASD group were significantly lower than that in the TD group. ASD, autism spectrum disorder; TD, typical developed group; DTI-ALPS, Diffusion Tensor Imaging along Perivascular Spaces; Mean DTI-ALPS, average DTI-ALPS index for both left and right hemispheres; L, left-hemispheric; R, right-hemispheric.
